# Microbiomes of built environments: 2011 symposium highlights and workgroup recommendations

**DOI:** 10.1111/j.1600-0668.2012.00782.x

**Published:** 2012-04-10

**Authors:** Richard L Corsi, Kerry A Kinney, Hal Levin

The air we breathe inside buildings dominates overall inhalation exposure to most air pollutants, whether of indoor or outdoor origin. The same is true for our exposure to microorganisms. Over the past three decades much has been learned about chemicals (in gas and particle phases) in building air, including typical levels, sources, fate, and control. Far less has been learned about the types, sources, and fate of microorganisms in buildings, and about how building design, and operation and maintenance affect microorganisms in buildings. Knowledge creation has been constrained by historical reliance on culture-based methods that can yield only partial or biased assessments of microbial community structure, sometimes dramatically underestimating uncultivable organisms, and failing to detect fragments of organisms that may themselves influence human health. However, in the past several years, advances in culture-independent analytical methods have significantly increased knowledge related to microbial communities and diversity in buildings. We are positioned to make even stronger gains in the coming years.

A 2-day symposium on microbiomes of built environments, sponsored by the Alfred P. Sloan Foundation, was held at Indoor Air 2011. The main goals were to review what has been learned about microbial communities in buildings over the past decades and to forge a vision of how culture-independent methods and tools might fill existing knowledge gaps in the future. The symposium included a keynote address (J. Craig Venter), state-of-knowledge and potential advancement summary presentations (Aino Nevalainen and Jonathan Eisen), and 15 podium presentations. The workshop involved more than 40 researchers who developed a list of 12 priority needs directed toward advancing the knowledge of microbial communities in buildings and the factors that affect those communities. We report here the recommendations that stemmed from this workshop.

- The interaction between microbial communities and building materials needs far greater attention. Building materials in occupied spaces and building envelopes are generally poorly characterized in terms of physical structure and chemical composition, factors that may influence the nature of microbial communities and growth rates.
- More attention should be given to longitudinal studies of microbial ecology in buildings. There is a need to examine how microbial communities change over time, particularly in response to changes in building environmental conditions, materials, and operation and maintenance practices.
- The sequencing of ‘reference genomes’ of cultured isolates of different kinds of microbes from the built environment would be a valuable community resource both for predicting functions of importance and for interpreting PCR and metagenomic sequence data.
- Future research should focus not just on the identification of microorganisms in buildings but also on their functioning. That is, we need to go beyond, ‘Who’s there?’ by also investigating, ‘What are they doing?’
- Shared research-building sites at several locations around the world would be highly beneficial for researchers. Having such sites would allow interdisciplinary researchers to conduct studies in a more controlled and systematic manner than is currently possible in field studies, where many of the building factors are uncontrolled (or not measured). These building test sites would enable researchers to test the performance of sampling methods and to assess how the indoor microbial communities respond to factors such as ventilation rates, thermal conditions, and human occupancy loads and activities. These sites should be located in different climatic zones and reflect the diversity of built environments.
- Given the importance of humans as sources of indoor bacteria, additional research is warranted to study the effects of human behavior and activity patterns on indoor bacterial communities. Such studies could focus not only on humans as sources, but also on how human activities such as cleaning affect microbial communities.
- Pets are important sources of indoor bacteria. More research is needed to understand their nature and significance. There is a need to increase the understanding of how the diet or cleaning frequency of an indoor pet affects it as a source of bacteria, and how bacterial communities vary for pets that spend time both outdoors and indoors as compared to pets that are always indoors.
- Research is needed to discern how microbiomes of built environments change as a result of climate change. Research should focus not only on direct impacts of climate change, for example, heat waves or dust storms, but also on changes in buildings to mitigate or adapt to climate change. Investigations of the latter factors should consider the effects of weatherization of existing buildings and of ‘tight’ envelopes of new buildings, increased use of green building materials, increased use of conventional and new insulation materials, and more.
- Researchers should have a checklist of metadata that merit collection during investigations of microbiomes in buildings. The list should include sampling methods, environmental conditions, information on ventilation and HVAC systems, building materials, building operation and maintenance practices, previous water challenges, and more. A consistent list used across research efforts will allow for greater comparison among studies.
- Research is needed to ascertain interactions between indoor microbial communities and indoor pollutants. For example, research is needed to determine whether carbon dioxide or ammonia leads to changes in the pH of water films on materials in such a way as to influence microbial growth or diversity, and whether products of indoor air chemistry or surface chemistry do the same.
- Consideration should be given to ‘citizen science’ projects for which the general population is involved in collecting samples that elucidate the nature of microbial communities in homes, classrooms, and other common indoor settings. Such an effort would require centralized analysis facilities. Metadata could be collected via questionnaire. One possibility is to use HVAC filters as common ‘sampling’ devices, which are donated to science instead of being discarded by homeowners or school-building staff after use.
- Development and verification of new technologies for routine surveillance of indoor microorganisms would facilitate field studies and, depending on cost and complexity, could be used for citizen science projects.

Molecular methods provide great opportunities to rapidly expand the existing knowledge base related to microbiomes of built environments. Recommendations stemming from this 2-day symposium at Indoor Air 2011 can provide a partial road map, guiding future research to better understand how building construction, operation, and maintenance affect indoor microbial communities.

